# Hemoglobin Mass and Blood Volume in Patients With Altitude-Related Polycythemia

**DOI:** 10.3389/fphys.2022.867108

**Published:** 2022-04-28

**Authors:** Walter F. J. Schmidt, Nadine Wachsmuth, Jesus Jimenez, Rudy Soria

**Affiliations:** ^1^ Department of Sports Medicine and Sports Physiology, University of Bayreuth, Bayreuth, Germany; ^2^ Instituto Boliviano de Biologia de Altura, Universidad Mayor de San Andres, La Paz, Bolivia

**Keywords:** chronic mountain sickness, hemoglobin concentration, plasma volume, carbon monoxide rebreathing, erythropoietin, peripheral oxygen saturation

## Abstract

Patients with chronic mountain sickness (CMS) have a high hemoglobin concentration [Hb] due to increased hemoglobin mass (Hbmass) and possibly reduced plasma volume (PV). The values of Hbmass, PV and blood volume (BV) have been described differently, and the relationships between [Hb] and Hbmass or PV are poorly understood. This study obtained representative Hbmass, PV and BV data from healthy, high-altitude residents and CMS patients and quantified the dependency of [Hb] on Hbmass and PV. Methods: Eighty-seven subjects born at high altitude (∼3,900 m) were enrolled. Thirty-four had CMS (CMS), 11 had polycythemia without CMS (intermediate, IM), 20 were healthy highlanders (HH), and 22 living near sea level (SL, 420 m) served as the sea level (SL) control group. Hbmass, PV and BV were determined using a CO-rebreathing method modified for assessing polycythemia patients. Furthermore, [Hb], hematocrit (Hct), plasma erythropoietin concentration [EPO] and blood gas and acid–base status were determined. Results: In the HH group, Hbmass was 27% higher (940 ± 105 g) than in the SL group (740 ± 112 g) and 72% (1,617 ± 265 g) lower than in the CMS group. The PV in the HH group was similar to that in the SL group (−6%) and 15% higher than that in the CMS group (*p* < 0.001). In the HH group, the BV (5,936 ± 673 ml) did not differ from that in the SL group and was 28% lower than in the CMS group (7,606 ± 1075 ml, *p* < 0.001). Log [EPO] was slightly increased in the CMS group relative to the HH group (*p* < 0.01). All values in the IM group were between those in the HH and CMS groups. Hbmass and BV were positively correlated, and PV was negatively correlated with peripheral O_2_ saturation. Increased Hbmass and decreased PV contributed approximately 65 and 35%, respectively, to the difference in [Hb] between the HH (17.1 ± 0.8 g/dl) and CMS (22.1 ± 1.0 g/dl) groups. Conclusions: In CMS patients, the decrease in PV only partially compensated for the substantial increase in Hbmass, but it did not prevent an increase in BV; the decrease in PV contributed to an excessively high [Hb].

## Introduction

Patients with chronic mountain sickness (CMS) or Monge’s disease (Monge 1942; Monge and Whittembury 1976) develop severe polycythemia, in which a hemoglobin concentration [Hb] of > 21 g/dl in men and > 19 g/dl in women is considered typical (Vasquez and Villena 2001; Leon-Velarde et al., 2005; Villafuerte and Corante 2016). The disease occurs at altitudes 2,500 m above sea level (SL) and above (Leon-Velarde et al., 2005; Villafuerte and Corante 2016), and it is assumed that peripheral chemoreceptors are desensitized. The altered respiratory stimulus leads to relative hypoventilation, which causes a reduction in the arterial O_2_ partial pressure and oxygen saturation of Hb (Severinghaus, Bainton, and Carcelen 1966; Leon-Velarde and Richalet 2006). This results in excessive erythropoiesis, which increases the number of red blood cells and blood viscosity (Kwaan and Wang 2003). Characteristic symptoms include vascular dysfunction (Rimoldi et al., 2012), decreased cerebral blood flow velocity (Claydon et al., 2005), increased pulmonary blood pressure (Maignan et al., 2009) (Soria et al., 2019) and heart failure, especially in the right ventricle (Leon-Velarde et al., 2005; Villafuerte and Corante 2016).

Although extremely high values for [Hb] and hematocrit (Hct) have been well documented as the main signs of the disease, there are limited and partly contradictory data on the factors underlying an excessive [Hb], i.e., the absolute hemoglobin mass (Hbmass) or red cell volume (RCV) and the plasma volume (PV). This information is also important in that [Hb] does not necessarily reflect the value of the Hbmass and PV consistently across ethnic groups. Thus, for example, the higher [Hb] of the high-altitude Andean people relative to the Sherpas from Tibet is not only due to a higher Hbmass but also to a lower PV (Stembridge et al., 2019). Additionally, the total blood volume (BV) in CMS patients has rarely been systematically examined; this parameter may contribute to severe complications characteristic of CMS, e.g., pulmonary hypertension (Soria et al., 2019) and right-heart failure as a result of possible chronic hypervolemia (Villafuerte and Corante 2016).

Of the abovementioned volumes, the RCV has thus far been the most studied. The classic studies by Lawrence et al. (1952) and Hurtado (1960) using radioisotope-based methods and Evans Blue dilution showed an increase of > 50% in healthy residents living at ∼4,500 m relative to SL residents and an additional increase of 10–40% in polycythemia patients. Recent studies generally confirmed an elevated RCV/Hbmass in CMS patients from similar altitudes, but the actual values differed greatly, ranging from 27 to 60% higher than that in healthy highlanders (HHs). Differences in the PV between CMS patients and HHs are even more controversial, ranging from no difference (Claydon et al., 2004; Oberholzer et al., 2020) to significantly lower values in CMS patients (approx. 30—40% lower) (Lawrence et al., 1952; Hurtado 1960). Similarly, data on BV differences are controversial, ranging from no difference between CMS patients and HHs to ∼25% higher values in CMS patients (Claydon et al., 2004; Oberholzer et al., 2020).

One possible reason for these different results is the methods of volume determination used in the different studies. Some studies determined PV using Evans Blue dye and calculated the RCV and BV *via* the [Hb] and Hct; others determined Hbmass/RCV directly using carbon monoxide or radioactive substances as tracers. Both methods have inaccuracies when calculating volume since the underlying cell factor, i.e., the ratio of the [Hb] in peripheral blood to the [Hb] in central blood, is not taken into account. Furthermore, the mixing time of the tracer used for Hbmass determination is prolonged in polycythemia patients relative to healthy subjects with a normal [Hb] (Wachsmuth et al., 2019), which when not taken into account leads to an underestimation of all volumes. It can therefore be stated that although all studies carried out thus far have shown an increase in the Hbmass/RCV of CMS patients, these data and the results for BV and PV have a certain level of inaccuracy.

The present study is part of a larger project. In the first recently published phase, the carbon monoxide (CO) rebreathing method was adapted to accommodate the special conditions of high-altitude residents and polycythemia patients (Wachsmuth et al., 2019). In the second phase, which is presented here, representative data for Hbmass and BV were collected from a large number of individuals before different therapeutic approaches for reducing Hbmass are evaluated in the upcoming third phase. Therefore, the first aim of the current study was to determine the total BV and the partial volumes of Hbmass/RCV and PV with a method specifically tailored to the patient cohort examined herein.

Until recently, an [Hb] > 19 g/dl in women and > 21 g/dl in men was considered to be the most obvious characteristic of CMS. In their recently published study on healthy and CMS-positive subjects in the world’s highest city (5,050 m), Oberholzer et al. (2020) demonstrated that, at least at that altitude, [Hb] has no additional diagnostic value for CMS. The reason for this finding might be that Hbmass and PV change in both HHs and CMS patients by unpredictable levels and thus influence the [Hb]. The second aim of this study was to determine the impact of Hbmass and PV on [Hb] in healthy and CMS-affected high-altitude residents and assess the validity and robustness of [Hb] as a diagnostic tool for CMS.

To differentiate the influences of normal chronic altitude adaptation from the pathophysiological effects occurring at higher altitudes, examinations were carried out in participants who were born in the same geographic area at an altitude of approx. 3,900 m. They either lived their whole life at this altitude or had been relocated to a lower altitude (∼400 m) for at least 2 years before the study was carried out and had completely adapted to the lower altitude during this time.

## Materials and Methods

### Ethical Approval

Ethical approval was granted by the ethics committee of the Universidad Mayor San Andres in La Paz, Bolivia (No. 846/2014). The study conformed to the standards of the Declaration of Helsinki, except for registration in a database. Written informed consent was obtained from all subjects. The subjects volunteered to participate in the study and were free to withdraw at any time without providing a reason.

### Subjects

In total, 87 male subjects who were born at altitudes of approximately 3,900 m participated in the study (Table 1). Thirty-four subjects were diagnosed with an [Hb] above 21 g/dl) and were included in the CMS group (Villafuerte and Corante 2016). Twenty healthy individuals who lived in the same environment as the CMS participants were included as a high-altitude-adapted control group (HH group). To differentiate between the effects of normal altitude adaptation and pathophysiological reactions, a second control group consisting of persons residing near SL (*n* = 22; SL group) was included. All members of this group were born at the same altitude as those in the CMS and HH groups but had emigrated to an area near SL at least 2 years before initiation of the study and lived in the city of Santa Cruz, Bolivia, (420 m) for 16.6 ± 11.2 years.

**TABLE 1:** Characteristics of study participants.

	Sea level residents *n* = 22	Healthy highlanders *n* = 20	Intermediates *n* = 11	CMS patients *n* = 34
Altitude of birth (m)	3,774 ±357	3,717±372	3,911±132	3,816±386
Altitude of residence (m)	420 ±0	3,827±85	3,975±121	3,950±124
Age (yrs)	41.4 ±14.2	44.5±12.7	49.8±14.1	55.3*±10.0
Height (cm)	168 ±8	165±6	163±6	165±6
Body mass (kg)	72.1 ±12.3	76.8±10.0	75.9±13.2	83.0±12.9
BMI (kg/m^2^)	25.7 ±4.5	28.3±3.5	28.4±4.2	30.5±4.2
LBM (kg)	52.8 ±5.1	53.2±4.9	51.4±6.5	55.1±5.2
Qinghai score	n.d.	n.d.	2.6±0.9	9.4±2.9

*p < 0.05.

Means ± standard deviations; chronic mountain sickness (CMS) patients, intermediates = originally suspected as CMS patients with actual [Hb] < 21 g/dl, healthy highlanders (HH), and sea level (SL) residents. LBM = lean body mass. n.d. = not determined. Significant differences with respect to the HH group.

Additionally, a fourth group of subjects was included (*n* = 11). These subjects were initially suspected to have CMS, but at the time when the study was conducted, their [Hb] did not exceed the cutoff value of 21.0 g/dl. Their [Hb] was, however, three standard deviations (STDs) above that of the HH control group, and they were therefore classified as the intermediate (IM) group. Exclusion criteria for participants in all subgroups were chronic lung diseases of various origins 3) and phlebotomy, also called sangria, within 3 months before the start of the study. All participants still living at altitude identified themselves as Aymara, while in the low-altitude group, eight participants were Aymara, twelve were Quechua and two were of Caucasian origin.

### Study Design

The study was carried out in the La Paz/El Alto area in Bolivia. Measurements of the subjects from this region were performed at the Instituto Boliviano de la Altura (IBBA) of the Universidad Mayor de San Andres, located at 3,600 m above SL. Measurements of the subjects from near SL took place in a commercial laboratory in Santa Cruz (420 m).

After the test subjects arrived at the laboratory, medical anamnesis—including the Qinghai score to assess the severity of CMS (Leon-Velarde et al., 2005; Villafuerte and Corante 2016)—was performed, followed by anthropometric measurements. After resting for 15 min in a sitting position, two tubes of cubital venous blood were drawn from the subjects and aliquoted. Hbmass was determined using the CO-rebreathing method modified according to Wachsmuth et al. (2019).

### Anamnesis, Anthropometric Determinations, and Analytic Methods

Medical anamnesis included questions about previous living conditions, previous illnesses and risk factors such as smoking and alcohol consumption. Body fat mass was determined with quadripolar impedance (Omron BF508 bioimpedance analysis, Omron, Osaka, Japan). The subjects were weighed, and the fat percentage and fat-free mass were determined in triplicate.

Two cubital venous blood samples, i.e., 9 ml of heparinized blood and 5 ml for serum analysis, were collected for determining basic hematological parameters as well as the erythropoietin (EPO) level. The whole procedure, including blood sampling, sample transport, sample storage and sample analyses, was performed under standardized conditions. Blood samples were collected after the subject rested for at least 15 min in a sitting position. All samples were analyzed in the laboratory of the IBBA, and those samples drawn at Santa Cruz were transported under cool conditions to La Paz within 24 h.

[Hb] was determined in triplicate using the cyanmethemoglobin method, and the Hct was determined using microcentrifugation. Serum EPO was measured by a solid phase, enzyme-labeled chemiluminescent immuno-metric assay (IMMULITE 1000 EPO; Siemens Healthcare Diagnostics, Llanberis, United Kingdom; intra-assay CV 4.2%). Prior to the CO-rebreathing procedure, capillary blood samples were collected from a hyperemic earlobe for analysis of blood gases and acid–base status. O_2_ partial pressure (PO_2_), CO_2_ partial pressure (PCO_2_), peripheral O_2_ saturation (SpO_2_) and pH were measured (i-STAT one Analyzer (Abbott, New Jersey, United States; OSM3, Radiometer, Copenhagen, Denmark), and the standard bicarbonate and base excess were calculated from these values.

### Hbmass and Blood Volume

For Hbmass and BV determination, a modified CO-rebreathing method, originally described by Schmidt and Prommer (2005) and Prommer and Schmidt (2007), was performed. A CO bolus was inhaled and rebreathed with pure oxygen for 2 minutes. Hbmass was then calculated from the difference in HbCO concentration before and after rebreathing. Essential prerequisites for accurate results are as follows: 1) knowledge of the amount of CO diffused into the blood; 2) complete mixing of the diffused CO, which leads to its homogeneous distribution in the blood; 3) knowledge of the volume of CO diffused from the blood into the tissue, i.e., mostly to myoglobin; and 4) knowledge of the volume of CO exhaled after removing the spirometer from the subject’s mouth.

For healthy test subjects who reside at SL, all of these factors are known or can be easily determined, so the increase in the HbCO concentration in blood 7 min after the start of the rebreathing procedure was used to calculate the Hbmass. In some patient groups, however, the CO uptake through breathing and the CO mixing time in the blood potentially differ. We determined these factors in CMS patients in a previous study and found that the mixing time of CO was prolonged (Wachsmuth et al., 2019), resulting in a HbCO plateau between the 14th and 20th minute after the start of rebreathing. Based on these results, we therefore determined the HbCO concentration for that period of time and adjusted all the other factors, i.e., CO diffusion to myoglobin and CO exhalation, to calculate Hbmass. After this modification was implemented, valid and reliable results were obtained, as reflected in a typical error (TE) of 1.6%. For a detailed description of the method, see Wachsmuth et al. (2019).

### Procedure Practice

To accustom the test subjects to the rebreathing procedure, the breathing maneuver was first practiced without CO inhalation before the final CO-rebreathing test was administered. The volume of CO to be inhaled was 0.9 ml per kg of body weight at low altitude. Because of the lower air density in La Paz (approximately 495 mmHg) and the higher [Hb] of the test subjects, the CO-volume was increased to 1.7 ml/kg for healthy high-altitude residents and to 2.0 ml/kg for IM and CMS patients. Since many of the patients were overweight or obese, the body mass for calculating the applied CO was adjusted to a theoretical body mass index (BMI) of 25. Similar to the method established by Schmidt and Prommer (2005), CO inhalation took place over 2 min. Blood samples were collected in triplicate from a hyperemic earlobe before inhalation and—different from the established method—every 2 min between the 14th and 20th minute after the start of inhalation. The mean value of these four samples was computed to calculate the HbCO difference induced by CO inhalation.

### Calculation of Hbmass, Blood Volume and Plasma Volume

Hbmass was calculated as described by Wachsmuth et al. (2019). RCV, BV and PV were calculated according to formulas 1–3.

RCV (ml) = Hbmass (g)/[Hb](g/dl) * Hct (1)

BV (ml) = Hbmass (g) * 100 / [Hb](g/dl)/ cf (2)

PV (ml)= BV (ml)- RCV(ml) (3)

where cf = cell factor (body/venous Hct ratio). Because the cf changes at different altitudes, we used the values of 0.91 for the SL group, 0.93 for the HH group, and 0.96 for the IM and CMS groups (Chaplin Jr. et al., 1953; Sanchez et al. 1970).

### Statistical Analysis

For statistical analysis, IBM SPSS version 25 software was used. The alpha level was set to 0.05. The normality of the distribution of the data was assessed with the Kolmogorov–Smirnov test. All parameters except the EPO level in the CMS group were normally distributed. To compare the mean anthropometric, hematological and acid–base data of the different groups, one-way ANOVA followed by the Bonferroni post hoc test was applied. In all these cases, the HH group was considered the reference group, and the differences in all values between the other groups and the HH group were calculated to determine significance. The EPO level was log-transformed, and the Mann–Whitney U test was applied as a post hoc test to assess the differences between the CMS group and HH group.

Linear regression analyses were performed including all participants as well as for all subgroups, with hematological and acid–base data as dependent variables and peripheral oxygen saturation as an independent variable. Additionally, bivariate linear and quadratic regression analyses were performed also including all participants as well as for all subgroups, with [Hb] as a dependent variable and Hbmass and PV as independent variables.

## Results

The altitude of birth of all test subjects was approximately 3,900 m, and all high-altitude residents lived continuously at this altitude. All the high-altitude groups tended to be overweight, and the CMS patients tended to be obese. The Qinghai scores were 9.6 ± 2.9 in the CMS group and 2.6 ± 0.9 in the IM group, indicating a moderate CMS status in the CMS group and a negative CMS status in the IM group.

Compared to that in the SL group, the PCO_2_ in the HH group was clearly decreased, while that in the CMS group was not different. The SpO2 was significantly decreased in the HH group (−7.2%), with more pronounced decreases in the IM (−11.7%) and CMS groups (−15.8%; Table 2). Additionally, compared with those in the SL group, HCO_3_- and base excess were decreased in the HH group and, to a lesser extent, the CMS group (Table 2).

**TABLE 2:** Blood gas and acid-base status.

	Sea level residents *n* = 22	Healthy highlanders *n* = 20	Intermediates *n* = 11	CMS patients *n* = 34
pH	7.43±0.02	7.43±0.03	7.42±0.03	7.39***±0.03
PCO_2_ (mmHg)	38.2***±2.6	31.9±1.9	35.1**±2.0	37.5***±2.9
PO_2_ (mmHg)	81.9***±9.6	54.5±3.8	49.0±5.0	45.2***±4.0
SpO_2_ (%)	96.0***±1.5	88.9±2.4	84.4**±4.5	80.3***±4.2
BE (mmol/L)	1.0***±1.7	−3.2±1.8	−2.1±1.2	−2.4±1.9
HCO_3_^−^(mmol/L)	25.3**±1.6	21.1±1.5	22.5±0.9	22.7**±1.8

**p < 0.01.

***p < 0.001.

Means ± standard deviations; chronic mountain sickness (CMS) patients, intermediates = originally suspected as CMS patients with actual [Hb] < 21 g/dl, healthy highlanders (HH), and sea level (SL) residents. PCO_2_ = CO_2_ partial pressure, PO_2_ = O_2_ partial pressure, SpO_2_ = peripheral O_2_ saturation, BE = base excess, HCO_3_^−^ = standard bicarbonate concentration; LBM = lean body mass. Significant differences with respect to the HH group.

**FIGURE 1:**
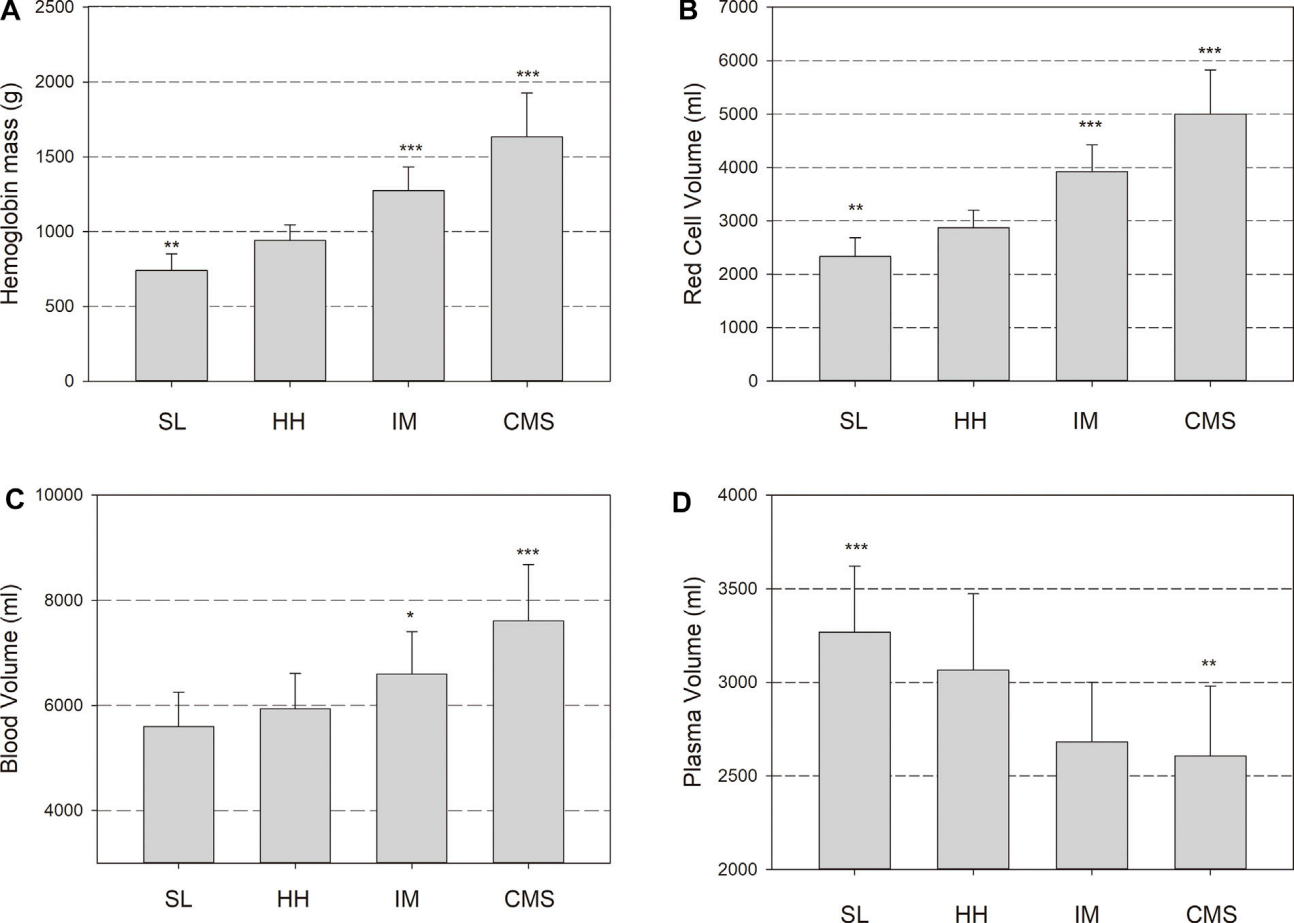
Hemoglobin mass (Hbmass), **(A)**, red cell volume (RCV), **(B)**, blood volume (BV), **(C)** and plasma volume (PV), **(D)** in sea level (SL) residents, healthy highlanders (HH), intermediate polyglobulic subjects (IM), and patients with chronic mountain sickness (CMS). Significant differences with respect to the HH group: * = *p* < 0.05, ** = *p* < 0.01, *** = *p* < 0.001.

Hemoglobin concentration [Hb] and the Hct were lower in the SL group than in the HH group and were clearly increased in the IM and CMS groups (Table 3). In the HH group, the Hbmass was 27% (940 ± 105 g) higher than that in the SL group (740 ± 112; *p* < 0.001) and 72% lower than that in the CMS group (1,617 ± 265 g, *p* < 0.001, Figure 1). Percentage differences of the same order of magnitude persisted when Hbmass was normalized to lean body mass (LBM) (Table 3). For the absolute and normalized RCV, similar percentage differences were observed between the groups (Figure 1 and Table 3).

**TABLE 3:** Hematological data, blood volumes, and serum EPO concentration.

	Sea level residents n = 22	Healthy highlanders n = 20	Intermediates n = 11	CMS patients n = 34
[Hb] (g/dl)	14.5***±0.9	17.1±0.8	20.1***±0.4	22.1***±1.0
Hematocrit (%)	45.6***±2.7	52.1±2.7	61.8***±1.3	68.4***±3.6
Hbmass (g)	740**±112	940±105	1,274***±158	1,617***±265
Hbmass (g/kg LBM)	14.1***±1.9	17.8±2.3	25.4***±2.5	29.3***±3.8
RCV (ml)	2,332*±353	2,871±327	3,918***±505	5,001***827±
RCV (ml/kg LBM)	44.3**±6.0	54.3±7.3	77.9***±7.3	90.7***±12.4
BV (ml)	5,599±657	5,936±673	6,599*±809	7,606***±1,075
BV (ml/kg LBM)	106.4±11.0	109.9±13.2	122.9*±12.3	125.2***±12.4
PV (ml)	3,268±353	3,066 407±	2,681*±320	2,605***±374
PV (ml/kg LBM)	62.1±6.0	57.8±7.3	54.2±6.0	47.2***±4.9
log [EPO] (mU/ml)	0.93±0.14	1.07±0.10	1.18±0.34	1.39**±0.43

*p < 0.05.

**p < 0.01.

***p < 0.001.

Means ± standard deviations; chronic mountain sickness (CMS) patients, intermediates = originally suspected as CMS patients with actual [Hb] < 21 g/dl, healthy highlanders (HH), and sea level (SL) residents. [Hb] = hemoglobin concentration, Hbmass = hemoglobin mass, RCV = red cell volume, BV = blood volume, PV = plasma volume, [EPO] = plasma erythropoietin concentration, LBM = lean body mass. Significant differences with respect to the HH group.

The absolute BV tended to be elevated by 6% in the HH group relative to the SL group (*p* = 0.1); in the CMS group, it was increased by 28% (*p* < 0.001; Figure 1). In the IM group, the BV was between that in the HH and CMS groups, i.e., 11% higher than that in the HH group (*p* < 0.05; Figure 1). The percentage differences were slightly less when normalized to LBM (Table 3). The PV tended to be smaller in the HH group than in the SL group (6%, *p* = 0.09) and was 15% smaller in the CMS group than in the HH group (*p* < 0.001; Figure 1 and Table 3).

Log [EPO] did not differ between the SL group and the HH group and was significantly higher in the CMS group than in the SL group (*p* < 0.01; Table 3).

### Regression Analyses

**FIGURE 2:**
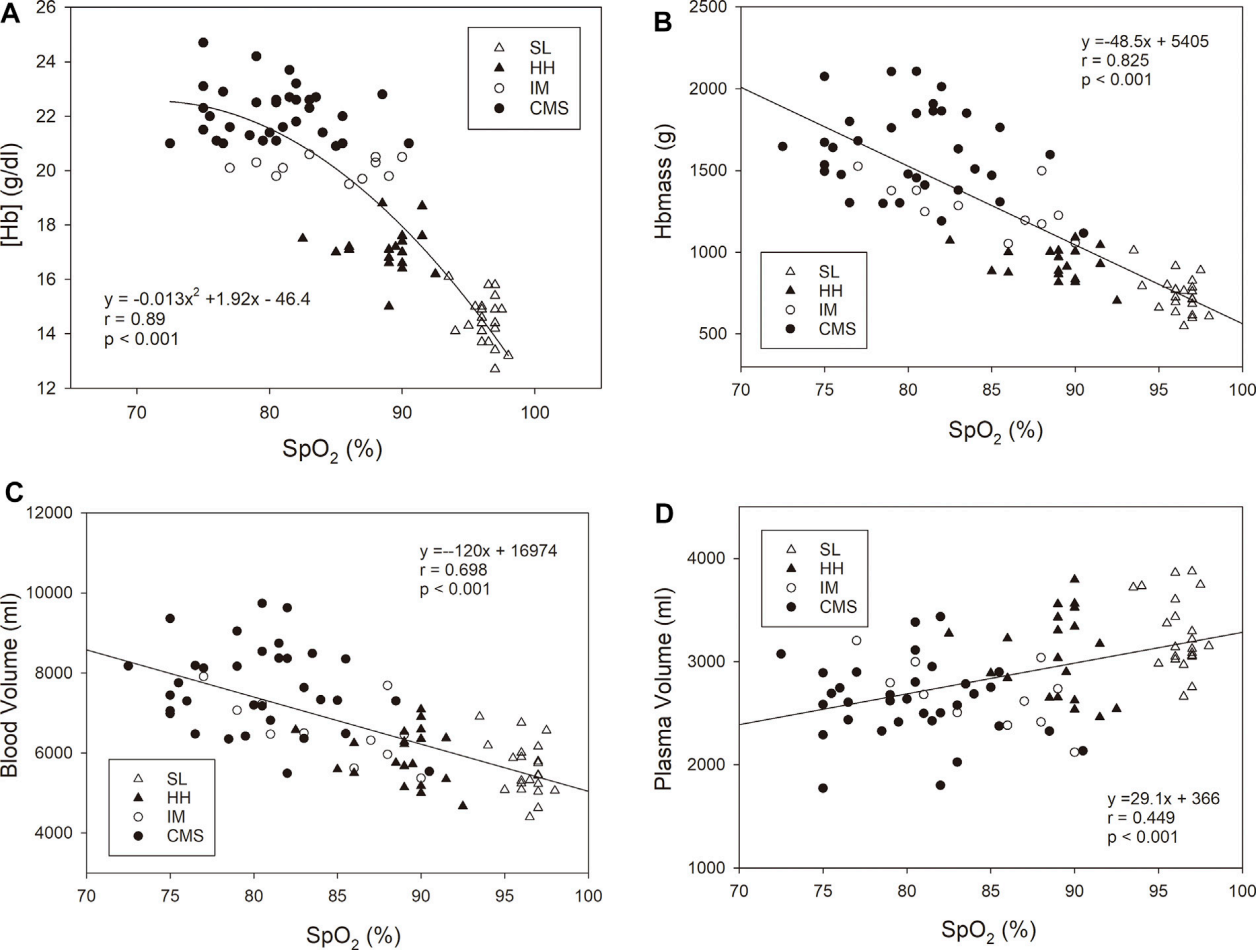
Oxygen saturation in arterialized blood (SpO_2_) vs. hemoglobin concentration [Hb], **(A)**, hemoglobin mass (Hbmass), **(B)**, blood volume **(C)**, and plasma volume **(D)**. The statistical information relates to the entire cohort. The regression analyses within the subgroups did not yield any significant relationship.

When all the subgroups were included in the analysis, the association of [Hb] with SpO_2_ was highly significant, with a very low scatter at a high SpO_2_ that increased with decreasing SpO2. While there were significant positive linear relationships between Hbmass and SpO_2_ and between BV and SpO_2_, there was a negative linear relationship between PV and SpO_2_ (Figure 2). The *p* value of the dependence of Hbmass, BV and PV on SpO_2_ was of the same order of magnitude when these variables were normalized to LBM. Log [EPO] was inversely associated with SpO_2_ (r = 0.576, *p* < 0.001). When the same regression analyses were performed within the subgroups, no significant associations were found.

**FIGURE 3:**
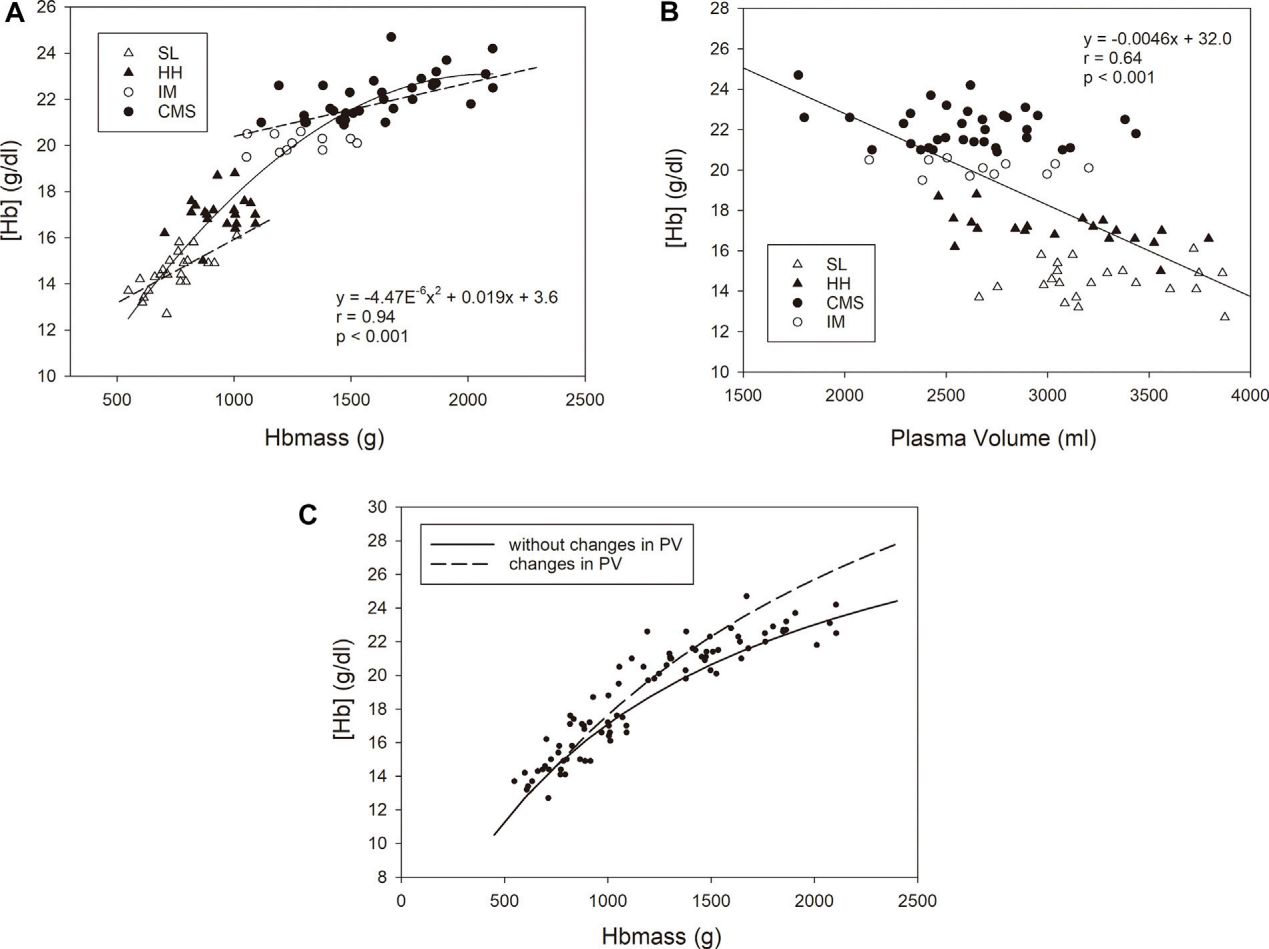
Relationship between [Hb] as the dependent variable and Hbmass **(A)** and plasma volume **(B)** as independent variables. The statistical information in the graphs relates to the group as a whole. For Hbmass vs. [Hb], significant associations were found for the CMS group (y = 0.0023x + 18.4 r = 0.63, *p* < 0.001) and the SL group (y = 0.0053x + 10.5, r = 0.69, *p* < 0.001). **(C)** Relationship between [Hb] and absolute Hbmass calculated for two conditions with respect to the SL group: **(i)** a constant plasma volume with increasing Hbmass (i.e., 3,300 ml, corresponding to the mean value of the SL group); **(ii)** a decreasing plasma volume with increasing Hbmass (dashed line). A reduction in PV of 80 ml per 100 g Hbmass above an Hbmass of 740 g (corresponding to the mean value of the SL group) was assumed. The reduction in PV is calculated from the average decrease in PV of all three altitude groups relative to the SL group. The dots represent the values determined in this study.

Figure 3 shows significant dependencies of [Hb] on Hbmass and PV in the whole group. While the [Hb] increased with a sigmoid-like function with increasing Hbmass, there was a negative linear relationship between PV and [Hb]. Among the subgroups, a significant correlation was only found for Hbmass vs. [Hb] in the SL and CMS groups. The multiple regression analysis with [Hb] as the dependent variable and Hbmass and PV as independent variables yielded highly significant main effects (*p* < 0.001) for Hbmass and PV and a significant interaction effect between both variables (*p* < 0.01).

## Discussion

To the best of our knowledge, the present study is the first to use a method specifically tailored to CMS patients to determine Hbmass and individual blood volumes. The most important results were a 27% higher Hbmass in the HH group relative to the SL group and only small changes in PV and BV. Among CMS patients, Hbmass increased by 72%, BV increased by 28% and PV decreased by 15% relative to the HH group. All of these changes were linearly correlated with a decrease in SpO_2_. [Hb] was positively correlated with Hbmass and negatively correlated with PV; however, a very high [Hb] was only weakly associated with Hbmass.

The blood gas and acid–base data indicated well-known altitude adaptations in the HH group in the form of metabolically compensated respiratory alkalosis (e.g., Wachsmuth et al., 2013), whereby the SpO_2_ was moderately decreased by 7.2% (Table 2). As demonstrated by the unchanged PCO_2_, compared to the SL group, the CMS group did not show an adequate hypoxic respiratory response, and the SpO_2_ decreased significantly by 8.6% with respect to that in the HH group. This behavior is well documented in the literature and is attributed to the desensitization of chemoreceptors, which is described in detail by León-Velarde and Richalet (2006) and Severinghaus et al. (1966). The PCO_2_ and SpO_2_ values in the IM group were between those in the CMS and HH groups and indicate a less pronounced reduction in the chronic hypoxic respiratory response.

### Hbmass and Red Cell Volume

Most studies investigating the effects of high altitude on Hbmass and BV in healthy volunteers and CMS patients were carried out in the Peruvian regions of Morococha and Cerro de Pasco at altitudes of approximately 4,350 m–4,540 m; accordingly, these studies tended to report hematological changes more pronounced than those in our study, which examined subjects who lived at approximately 4,000 m. Both classic studies that used either radioactive tracers for red blood cell labeling (Lawrence et al., 1952) or Evans blue dye (Hurtado 1960) showed an increase in RCV by approx. 50—60% in healthy high-altitude residents, which is much higher than our corresponding finding (+23%; Table 3), while (Oberholzer et al., 2020) showed a similar result (+17%) in residents at the same altitude as that of our study. Additionally, Claydon et al. (2004) and Hansen et al. (2021) reported absolute data similar to those in our study in high-altitude residents, but unfortunately, they did not present data for a SL control group. It is possible that intoxication with cobalt from the surrounding cobalt mines augments hypoxia-mediated erythropoiesis (Jefferson et al., 2002) and contributes to the excessive erythrocytosis described by Lawrence et al. (1952) and Hurtado (1960).

The pathophysiological effects of CMS described by Lawrence et al. (1952) (+48%), Hurtado (1960) (+85%), Claydon et al. (2004) (+58%) and Oberholzer et al. (2020) (+47%) were relatively similar to those described in our study (+67%), but the effect was considerably smaller in the study by Hansen (+27%). This may be due to methodological reasons, i.e., an inappropriate tracer (CO) mixing time, which was only 7 min. In our recent methodological study, we determined that complete mixing occurred after 14 min. Assuming a mixing time of only 7 min results in an underestimation of 7%, which results in an underestimation in Hbmass of ∼110 g (Wachsmuth et al., 2019).

The physiological reason for the higher Hbmass at high altitudes is lower O_2_ availability; we showed a linear relationship between decreased SpO_2_ and increased Hbmass (Figure 2). However, the increased scatter of Hbmass with decreasing SpO_2_ indicates a difference in the sensitivity of the erythropoietic system to prevailing hypoxia in the CMS group. For these individuals, a mean SpO_2_ of 80% was associated with an Hbmass value between ∼1,250 g and ∼2,150 g, corresponding to 23 g/kg LBM and 35 g/kg LBM. The lack of significance in the relationship between Hbmass and SpO_2_ within the CMS group can be similarly interpreted. In addition to methodological influences when determining SpO_2_ (see limitations), another cause may be the large scatter in the individual erythropoietic reactions of the patients to the lower oxygen content in the arterial blood.

The mechanism by which Hbmass is increased at high altitudes is generally assumed to be due to a higher [EPO]. This is the case for short stays at high altitude, where the [EPO] reaches its maximum around the second day (e.g., Heinicke et al., 2003). Subsequently, however, it drops significantly, and as also demonstrated in this study, no difference between HH and those residing at SL can be identified despite a significantly higher Hbmass (Heinicke et al., 2003). In the CMS group in our study, log [EPO] was slightly but significantly higher than in the HH group (Table 3). This is in accordance with the data from the study by Villafuerte et al. (2014), who distinguished between two groups of patients: one with an [EPO] similar to that of those at SL and the other with an elevated [EPO]. Even though there was a slightly significant correlation between log [EPO] and SpO_2_ in this study, the increase in [EPO] was notably less than expected given the substantially increased Hbmass. This applies in particular to the IM group, who despite presenting with 43 and 81% increases in Hbmass relative to those in the HH and SL groups, respectively, did not show a significant increase in [EPO]. This lack of significance is possibly due to the high interindividual scatter of the serum [EPO]. However, physiological effects such as altered EPO availability as a result of a lower EPO receptor concentration in the serum could also contribute to these observations (Villafuerte et al., 2014).

### Plasma Volume and Blood Volume

While data on RCV are relatively homogenous among available studies, changes in PV differ widely. In our study, PV tended to be 6% lower in the HH group than in the SL group. While the classic studies (Lawrence et al., 1952; Hurtado 1960) and also Hansen et al. (2021) and Claydon et al. (2004) showed that high-altitude values were similar to sea-level values, Oberholzer et al. (2020) found 28% lower PV values in residents at 3,800 m than in control subjects at SL. For CMS patients, the classic studies (Lawrence et al., 1952; Hurtado 1960) found a marked reduction in PV by approximately 40%, which was much lower than those described by Hansen et al. (2021) (−16%) and Claydon et al. (2004) (−9%, nonsignificant). Oberholzer et al. (2020) did not find any difference between the HH and CMS groups. In our study, PV was 15% lower in the CMS group than in the HH group, corresponding to the data from Hansen et al. (2021).

In the literature, the changes in BV at an altitude of approx. 4,000 m are very inconsistent and range from −9% (Oberholzer et al., 2020) to +28% (Hurtado 1960) in HHs relative to SL residents; only a small, nonsignificant difference (6%) was found in our study. For CMS patients, who in our study showed a BV 28% higher than that in the HH group, a volume increase is mostly reported in the literature, ranging from 10 to 40% (Lawrence et al., 1952; Hurtado 1960; Oberholzer et al., 2020). Only Hansen et al. (2021) did not find a difference.

The reason for these discrepancies may at least partly be due to methodological differences, as some studies used red cell or Hb labeling and calculated PV and BV by means of the prevailing [Hb] and the hematocrit, while others determined PV directly by using Evans Blue dye. When calculating BV and PV using substances that label Hb or erythrocytes, a so-called cell factor of 0.91 (i.e., the ratio of Hct in peripheral blood to that in central blood) is usually applied (Chaplin Jr. et al., 1953). This cell factor varies among individuals and is influenced by factors such as posture, various diseases and altitude (Sanchez et al., 1970; Busk et al., 2017).

To our knowledge, none of the aforementioned studies that determined BV and PV at high altitude considered the body/venous Hct ratio and its possible variation between SL and high altitude. In the only available study that determined RCV and PV simultaneously at high altitude (4,540 m) by using Fe^52^ and Even’s Blue as markers, Sanchez et al. (1970) found a significantly increased cell factor (cf. = 0.96) in high-altitude residents, including CMS patients, compared to SL residents (cf. = 0.91).

We applied these considerations to our study and calculated PV with the respective cell factors. As a result, the PV values were lower by 420 ml in the CMS group, 360 ml in the IM group and 130 ml in the HH group than the values that did not consider these correction factors. Although there is certainly individual scatter, the values calculated here should be closer to reality than if the altitude-related cell factors had not been taken into account. Our data therefore allowed us to quantify the effects of the increased RCV and decreased PV in CMS patients on the difference in BV between the CMS and HH groups. Compared to the HH group, on average, RCV was increased by 2,130 ml in CMS patients, while PV was decreased by 460 ml. The effect of the increased RCV is thus much greater than that of the reduced PV, resulting in a mean BV increase of 1.7 L in CMS subjects from this study. The reduction in PV is therefore by far unable to compensate for the increase in RCV, which is in contrast with the findings of Hansen et al. (2021) but in accordance with all of the other findings on BV in CMS patients.

Concerning the underlying physiological mechanisms for the changes in PV, acute exposure to high altitude results in a rapid decrease in PV of ∼21% after 7 days at 4,000 m (Beidleman et al., 2017) due to the hypoxia-mediated suppression of the renin–aldosterone axis and modulation of serum atrial natriuretic peptide (ANP) (Siebenmann et al., 2015). This hormonal response is weak but still occurs in healthy high-altitude residents (Schmidt et al., 1999). In CMS patients, however, the suppression of the renin–aldosterone axis is highly pronounced and is probably an important reason for the decreased PV in CMS patients (Steele et al., 2021). Whether the increased BV in CMS patients is counterbalanced by baroreflex-mediated lower plasma antidiuretic hormone (ADH) and higher ANP concentrations (Schmidt et al., 1999) remains unknown.

### Hemoglobin Concentration

The [Hb] and the Hct are the best indicators of CMS and are routinely used for the diagnosis of the disease. The normal value for male residents at ∼4,000 m is 17.3 ± 1.5 g/dl (Vasquez and Villena 2001), which is in accordance with our data and has been confirmed in numerous studies. As in our study, mean values between 22 g/dl and 23 g/dl and extreme values of up to 25 g/dl have been described in CMS patients. Recently, however, Oberholzer et al. (2020) showed that the threshold value of 21 g/dl is no longer meaningful at very high altitudes of approximately 5,000 m, since it is exceeded in both CMS patients and CMS-negative persons.

This finding prompts the following question: to what extent does [Hb] provide only limited information at lower altitudes? Since [Hb] is a relative value and is determined by both Hbmass/RCV and PV, both of which are subject to opposite changes at altitude, we calculated the contributions of both parameters to [Hb]. As shown in Figure 3A, there was a very close (r = 0.94) positive but nonlinear relationship between Hbmass and [Hb], indicating that the increase in Hbmass does not continuously reflect changes in [Hb], e.g., an increase in Hbmass from 500 to 1,000 g is accompanied by an increase in [Hb] of 5.9 g/dl, but an increase from 1,500 g to 2000 g is accompanied by an increase in [Hb] of only 1.3 g/dl. Very similar, albeit somewhat smaller, differences in the relationship between Hbmass and [Hb] become evident when the increase in [Hb] is calculated due to altitude effects and to the severity of CMS disease. When calculating the altitude effects by including just the SL- and HH-groups in the regression analysis, an increase in Hbmass by 500 g was associated with an increase in [Hb] by 4.0 g/dl. Including only the CMS group in the regression analysis yielded an increase in [Hb] by 1.1 g/dl when Hbmass increased from 1,500 to 2000 g. Conversely, a change in [Hb] in this high range is also not an accurate indicator of the extent of erythropoiesis. This is partly due to PV being negatively associated with [Hb], as a 500 ml decrease in PV was accompanied by a 2.3 g/dl increase in [Hb] (Figure 3B). Therefore, the increase in Hbmass and the decrease in PV have synergistic effects on [Hb].

In Figure 3C, the calculated trajectory of [Hb] with increasing Hbmass is shown, assuming a constant PV (as in the SL group) or a changing PV (as was found in this study in the three high-altitude groups). At an Hbmass of 1,620 g, which was the mean in the CMS group in our study, an observed decrease in PV by ∼670 ml relative to that at SL resulted in an additional 1.8 g/dl increase in [Hb], i.e. ∼35% of the difference between the CMS and HH groups. However, the extent to which PV can decrease is limited. This is demonstrated by the real [Hb] values in this study, which increased in a saturation function and nearly plateaued at very high Hb levels (see Figure 3C). Thus, the judgment of the severity of polycythemia is rather imprecisely represented by [Hb] in the extreme ranges.

The decrease in PV contributes to the increase in [Hb] and thereby probably to the deteriorated rheological properties of blood in CMS patients (Steele et al., 2021). On the other hand, it counteracts the hypervolemia caused by the substantial increase in RCV. Even in healthy high-altitude dwellers, PV seems to play a potentially important role in the adaptation to hypoxic conditions. For example, Sherpas who have adapted to hypoxic conditions for a longer period of time have a greater PV than Andean residents who have probably lived at higher altitudes for a shorter time (Stembridge et al., 2019). A reduction in PV, as occurs in CMS patients, could thus be interpreted as a relatively short-term adjustment to the lower O_2_ availability. It is, however, not yet possible to assess whether a reduction in PV can be viewed as positive or negative regarding the course of the disease.

### Hypervolemia

The scatter of the [Hb], Hbmass and BV values were very similar in both control groups, implying that chronic hypoxia causes very uniform adaptation in healthy subjects. In contrast, CMS patients do not represent a homogeneous group. The STD for Hbmass in the CMS group was approximately 2.5 times higher than that in the HH group, and the STD for BV was also increased by ∼50%. The 90% percentile of BV in the CMS group was 9.2 L, which was 3.2 L above normal values in the SL and HH groups. Extreme hypervolemic stress can therefore be assumed in severe cases of polycythemia.

In their excellent review article, León-Velarde et al. (2010) described the effects of increased BV in CMS patients as very diverse. The increased BV compensates for the reduced venous return to the heart caused by increased viscosity, which results in normal cardiac output (Q). The increased BV also has a generally positive effect on the orthostatic reaction, which is improved in CMS patients relative to HH controls (Claydon et al., 2004). On the other hand, increased central venous pressure not only increases systemic diastolic pressure but also may contribute to an increase in pulmonary arterial pressure (León-Velarde et al., 2010), which may lead to right-heart failure. Additionally, it is not yet clear whether or to what extent hypervolemia amplifies the effects of chronic hypoxic pulmonary vasoconstriction. Further studies on the possible importance of hypervolemia in the development of right heart failure in CMS patients are necessary.

### Intermediate- and Sea Level-Groups

In addition to the comparison of CMS patients and healthy high-altitude dwellers, as is usually the case when examining chronic mountain sickness, two other groups were included in this study. A special second aim was to examine the possible development from a healthy to a manifest pathological condition (IM group) and to show possible changes when the hypoxic stimulus disappears due to a descent to low altitudes (SL group).

In addition to genetic causes, which were not examined here, various factors, such as male sex, age and obesity (De Ferrari et al., 2014), are known to be associated with relative hypoventilation and frequent sleep apnea, which triggers excessive erythropoiesis via reduced arterial SO_2_ (Villafuerte and Corante 2016). With regard to all hematological parameters demonstrated here, the IM group lies between HH and CMS, so it could be considered a transition state between healthy and pathological status. When the three altitude groups were included in multifactor ANOVA, the independent variables age and BMI showed significant main effects on SpO_2_, PO_2_ and PCO_2_ as well as on Hbmass and PV. Although this analysis does not prove cause and effect, it does suggest that, in addition to age, obesity plays a significant role in the development of CMS (De Ferrari et al., 2014). In this context, it would be very interesting to examine in a long-term intervention study whether a reduction in BMI, especially in the IM group, is associated with an improvement in blood gas status and a normalization of Hbmass, PV and BV.

The SL group was included in the study to quantify the chronic hypoxia effects of the HH group without having to address ethnic differences, which would have been necessary when comparing the data with those of Caucasians deriving from sea level. However, the SL group was also not ethnically homogeneous and consisted of 40% Aymara and 60% Quechua. When comparing Hbmass between the two groups, the Aymara tended toward higher absolute (*p* = 0.10) and normalized (*p* < 0.017) Hbmass values. Similar to the different altitude adaptations of residents of Tibet and the Andes with higher Hbmass values in the Andes and larger PVs in Tibet (Stembridge et al., 2019), a different adaptation strategy could also exist within the Andean populations. However, it must be noted here that neither the Aymara nor Quechua represent a uniform ethnic group but can be described as mestizos due to the genetic mixture with the predominantly Spanish conquistadors.

Regardless, this study shows that the hematological altitude adaptations in Andean people whose ancestors have lived at high altitude for many generations and who have themselves migrated to the lowlands are only temporary, and their values have probably completely approached those of lowland Caucasians (Wachsmuth et al., 2019) in a relatively short time. Some of these changes take place within a few days, as shown by the increase in PV by approximately 500 ml in young healthy athletes half a week after the descent from La Paz (3,600 m) to Santa Cruz (400 m) (Wachsmuth et al., 2013). If lowland dwellers spend several weeks at higher altitudes, their Hbmass will return to the initial level within a few weeks after descent, with no neocytolysis taking place (Klein et al., 2021). It is, however, not known over what time course the Hbmass of CMS patients drops to lowland levels. Although their [Hb] decreases within a few weeks, to our knowledge, these are only reports of experience, and no scientific data are available. The examination of Hbmass and BV in CMS patients after descent to low altitudes could therefore provide important information for practice.

### Limitations

Although the altitude-dependent change in the ratio of the [Hb] in peripheral blood to the [Hb] in central blood volume (cf) was taken into account for the first time in the present study, inaccuracies in the calculation of PV and BV must be considered. Exact data can be obtained only if Hbmass/RCV and PV are determined simultaneously, each using direct methods. However, since it is no longer possible to use Evans Blue dye as a tracer for determining PV, the calculation applied here is probably the best solution.

In this study, Hbmass and BV were also normalized to LBM since the fat content varied greatly among groups and normalization to body mass would lead to incorrect conclusions. The LBM was calculated by measuring the fat mass using bioelectrical impedance analysis (BIA). Although this method has a relatively high level of inaccuracy, it correlates with the gold standard methods (e.g., dual-energy X-ray absorptiometry (Hofsteenge et al. 2015)), which are not available in Bolivia. The LBM-normalized hematological data show, however, almost the same significant differences between the individual groups as the absolute data, so that they can be assumed to be valid.

The regression analyses of this study were carried out for all participants and for the individual subgroups. There was a clear dependency of the hematological data on the SpO_2_ for the group as a whole, but there were no significant associations in the subgroups. In the case of SL and HH, this can be attributed to the homogeneous composition of the participants, but in the case of CMS, one would have expected clear relationships given the large individual scatter of the hematological values. Despite the standardized nature of the procedure, this missing association could possibly partly be due to inaccuracies in the determination of SpO_2_, since, for example, hyperventilation and differences in skin perfusion sometimes cannot be avoided especially at cold temperatures. Arterial measurements would be of great benefit here.

## Conclusions

The present study is the first to use a method specifically tailored to CMS patients to determine Hbmass and BV. Compared with the SL group, the HH group had a 27% higher Hbmass, and showed a tendency to 6% higher BV and 6% smaller PV. In the CMS group, these values were +72% for Hbmass, +28% for BV and −15% for PV relative to the values in the HH group. All of these changes were linearly correlated with a decreasing SpO_2_.

The substantial increase in [Hb] was due to both an increase in Hbmass (accounting for ∼65% of the increase) and a reduction in PV (accounting for ∼35%). If [Hb] is excessively high, its relationship to Hbmass is generally eliminated, and [Hb] is only an imprecise measure of the extent of polycythemia. Despite a reduced PV, the pronounced increase in BV in CMS patients leads us to expect substantial influences on the cardiovascular system.

## Data Availability

The raw data supporting the conclusions of this article will be made available by the authors, without undue reservation.
